# Molecular Epidemiology and Genetic Context of *optrA*-Carrying Linezolid-Resistant Enterococci from Humans and Animals in South Korea

**DOI:** 10.3390/antibiotics14060571

**Published:** 2025-06-03

**Authors:** Younggwon On, Sung Young Lee, Jung Sik Yoo, Jung Wook Kim

**Affiliations:** 1 Division of Antimicrobial Resistance Research, National Institute of Health, Korea Disease Control and Prevention Agency, Cheongju-si 28159, Republic of Korea; on0740@korea.kr (Y.O.); blueskyi7@korea.kr (S.Y.L.); jungsiku@korea.kr (J.S.Y.); 2 Division of Zoonotic and Vector-Borne Disease Research, National Institute of Health, Korea Disease Control and Prevention Agency, Cheongju-si 28160, Republic of Korea

**Keywords:** linezolid-resistant *Enterococcus*, *optrA*, antimicrobial resistance, whole-genome sequencing, One Health

## Abstract

**Objectives:** Linezolid resistance among *Enterococcus* species poses a growing clinical and public health concern, especially due to the dissemination of transferable resistance genes, such as *optrA*. This study aimed to evaluate the prevalence of linezolid resistance and to characterize the molecular epidemiology and genetic contexts of *optrA*-positive linezolid-resistant *Enterococcus* (LRE) isolates from clinical and animal sources in South Korea. **Methods:** A total of 2156 *Enterococcus* isolates, collected through nationwide surveillance from hospitalized patients and healthy livestock (pigs, cattle, and chickens) between 2017 and 2019, were retrospectively analyzed. Phenotypic susceptibility testing, *optrA* gene screening, and whole-genome sequencing were performed to investigate genetic environments and phylogenetic relationships. **Results:** The prevalence of linezolid resistance was 0.2% in clinical isolates, 3.3% in pigs, 4.3% in cattle, and 1.4% in chickens. *optrA*-positive linezolid-resistant isolates were less frequent, with rates of 0.1%, 1.4%, 0.9%, and 1.0%, respectively. Multilocus sequence typing identified sequence types (STs) 330 and ST476 in *E. faecalis* from humans, with no shared STs between human and livestock isolates. The *optrA* gene was located either chromosomally, frequently associated with transposon Tn6674, or on multidrug resistance plasmids. Notably, *optrA* variants exhibited host-specific distribution patterns. Phylogenetic analysis demonstrated considerable genomic diversity, and Korean ST476 isolates were genetically related to international strains reported from livestock, poultry products, and wild birds, suggesting potential global dissemination. **Conclusions:** This study provides a comprehensive, nationally representative assessment of linezolid resistance in South Korea. The findings highlight the zoonotic potential and possible international dissemination of *optrA*-carrying ST476 lineages, underscoring the need for integrated One Health surveillance to monitor and control the spread of transferable resistance genes.

## 1. Introduction

*Enterococcus faecalis* and *Enterococcus faecium* are important opportunistic pathogens in humans and animals. They cause a wide range of infections and contribute significantly to antimicrobial resistance in clinical and veterinary settings.

Vancomycin-resistant enterococci (VRE), in particular *Enterococcus faecium*, are major opportunistic pathogens due to the limited therapeutic options available for vancomycin resistance [1]. Linezolid, the first drug of the oxazolidinone family, is an alternative antibiotic that is used against VRE [2]. Florfenicol, a phenicol-class antibiotic, is used to control respiratory infections in livestock. However, acquired cross-resistance to linezolid and florfenicol has emerged in the past decade [3,4,5], posing a serious challenge in both human and veterinary medicine.

Linezolid resistance can be attributed to chromosomal mutations in the 23S rRNA gene (mainly G2576T and G2505A), or acquisition of *cfr*, *optrA*, and *poxtA*, which are often found on plasmids or as composite transposons [6,7]. *cfr* encodes a 23S rRNA methyltransferase that confers resistance to phenicols, lincosamides, oxazolidinones, pleuromutilins, and streptogramin A [5]. While it has mainly been described in animal staphylococci isolates, few clinical enterococci isolates have been reported [3,6]. *optrA* encodes an ABC-F protein that confers cross-resistance to oxazolidinones and phenicols and was first described in China in *E. faecalis* and *E. faecium* isolates of both human and animal origins [4]. *poxtA* also encodes an ABC-F protein that is associated with decreased susceptibility to phenicols, oxazolidinones, and tetracyclines [5]. These three genes confer cross-resistance to linezolid and phenicols and have been reported to co-locate with phenicol resistance genes, such as *fexA* and *fexB* [3,4]. The emergence of linezolid-resistant bacteria, such as methicillin-resistant *Staphylococcus aureus* and VRE, has been reported globally in both human patients and livestock. Although the incidence of linezolid resistance is low, reports about the transferable *optrA* gene are increasing [6,8]. The global dissemination of *optrA*-positive Enterococcus isolates has been increasingly documented in clinical, livestock, and environmental settings across Asia, Europe, and North America [9,10]. Notably, a recent large-scale study in China revealed a high prevalence and plasmidome diversity of *optrA*-carrying *E. faecalis* among healthy individuals, with genomic similarities to strains isolated from animals and humans [9]. In Europe, comparative genomic analyses have identified zoonotic transmission routes and shared *optrA* variants among isolates from humans, livestock, and wildlife, underscoring the gene’s broad ecological distribution and mobility [10].

Furthermore, the co-detection of *cfr*, *optrA*, and *poxtA* genes in isolates from both human and animal sources suggests that the clinical use of linezolid and the application of phenicols in livestock may contribute to the selection and maintenance of mobile linezolid resistance determinants. Therefore, clarifying the genetic contexts of these resistance genes, particularly those conferring resistance to critically important antimicrobials such as linezolid, is essential for understanding and limiting their spread, especially within a One Health framework.

Given the increasing detection of mobile linezolid resistance genes, particularly *optrA*, in both human and animal isolates, there is a growing need for comprehensive genomic surveillance to elucidate their dissemination pathways. In this study, we investigated the prevalence of mobile linezolid resistance genes in linezolid-resistant *E. faecalis* and *E. faecium* specimens isolated from humans and livestock through nationwide surveillance in South Korea between 2017 and 2019. Additionally, we characterized the genetic environments of these resistance genes using whole-genome sequencing and analyzed the phylogenetic relationships among the isolates.

## 2. Results

### 2.1. Prevalence and Antimicrobial Resistance of Linezolid-Resistant Enterococci

**Figure 1:**
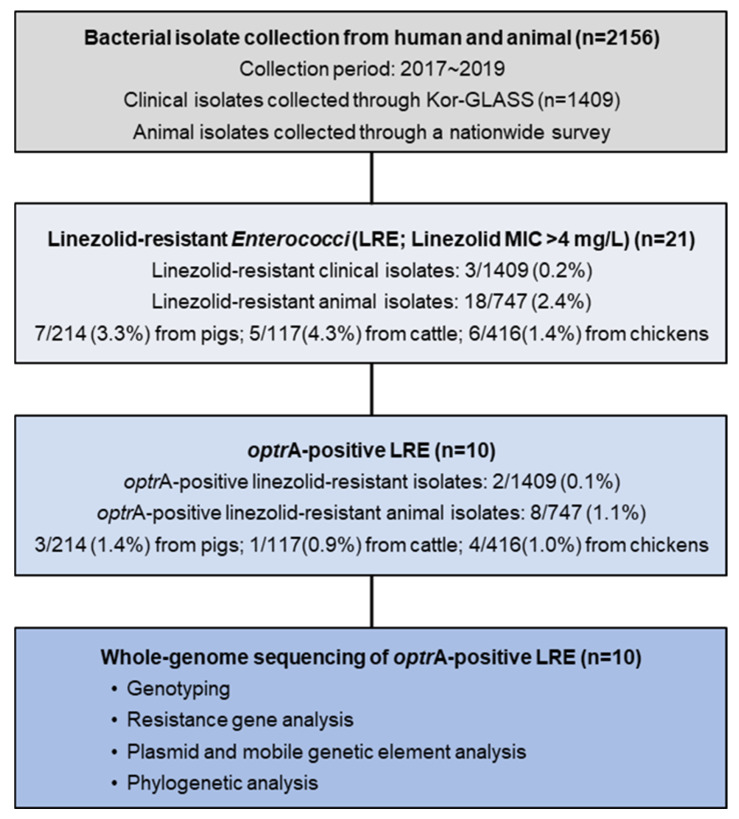
Overall study workflow illustrating the collection of *Enterococcus* isolates from humans and animals (pigs, cattle, and chickens), screening for linezolid resistance (MIC > 4 mg/L), detection of *optrA*-positive isolates, and selection for whole-genome sequencing (WGS). The number of isolates at each step is indicated.

Between 2017 and 2019, a total of 1409 clinical and 747 livestock Enterococcus isolates (pigs, n = 214; cattle, n = 117; chickens, n = 416) were collected. Among the clinical isolates, three (0.2%) *E. faecalis* strains were resistant to linezolid (two in 2017 and one in 2019). In livestock, linezolid resistance was detected in 18 isolates (2.4%), including 7 (3.3%) from pigs, 5 (4.3%) from cattle, and 6 (1.4%) from chickens, indicating a higher prevalence in livestock compared to in clinical isolates (Figure 1).

Among 21 linezolid-resistant Enterococci (LRE) isolates, acquired linezolid resistance genes were identified in 47.6% (10/21), all of which carried *optrA*. Three isolates from pigs harbored both *optrA* and *poxtA*, with a linezolid MIC > 16 μg/mL (Table 1). Additionally, seven of the ten *optrA*-positive isolates did not carry other known linezolid resistance genes or mutations, indicating that the resistance phenotype was attributable solely to *optrA*.

**Figure 2:**
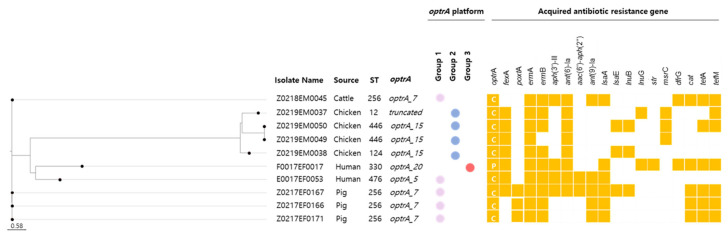
Heat map showing the presence or absence of acquired antimicrobial resistance genes and genetic features in ten *optrA*-positive *Enterococcus* isolates. Genes are grouped by antimicrobial class as follows: oxazolidinones (*optrA*, *poxtA*), macrolides (*ermA*, *ermB*, *lnuB*), aminoglycosides (*aac(6’)-Ie-aph(2")-Ia*, *ant(6)-Ia*, *aph(3’)-IIIa*), trimethoprim (*dfrG*), phenicols (*fexA*, *cat*), and tetracyclines (*tetM*, *tetL*). Each row represents an isolate; each column corresponds to a resistance gene. Yellow shading indicates the presence of a gene, and white indicates its absence. *optrA* variants are indicated within the *optrA* column for each isolate. Chromosomal (C) or plasmid (P) localization of *optrA* is also indicated. Isolates are also categorized into Group 1, 2, or 3 based on structural similarities in the *optrA* genetic environment identified through whole-genome sequencing.

All *optrA*-positive isolates remained susceptible to vancomycin, teicoplanin, daptomycin, and tigecycline. Chloramphenicol resistance (MIC > 16 mg/L) was detected in nine isolates, and one isolate exhibited intermediate susceptibility (MIC = 16 mg/L) (Table 1). The *fexA* gene was identified in seven isolates, two of which also carried the *cat* gene (Figure 2).

### 2.2. Molecular Typing and Genetic Characteristics of optrA-Positive Isolates

Multilocus sequence typing (MLST) assigned *E. faecalis* isolates to three sequence types (STs): ST330 and ST476 among clinical isolates, and ST256 among livestock isolates from pigs and cattle. In *E. faecium*, which was isolated exclusively from chickens, ST12, ST124, and ST446 were identified (Table 1).

Comparison of *optrA* nucleotide sequences with the reference gene (GenBank accession no. KP399637) identified four variants: *optrA_5* and *optrA_20* in clinical isolates, *optrA_7* in pigs and cattle, and *optrA_15* in chickens (Supplementary Figure S1). These variants were not restricted to specific STs or host origins, suggesting multiple independent acquisition events or horizontal gene transfer across genetically diverse backgrounds.

Analysis of the genetic context revealed that *optrA* was located either on the chromosome or plasmids, as determined by its assembly structure and the presence of replication-associated genes. Notably, chromosomal integration was observed in nine isolates—five *E. faecalis* (ST256 and ST476) and four *E. faecium* (ST12, ST124, and ST446)—whereas a single clinical isolate carried *optrA* on a plasmid. This distribution highlights the dual mechanisms of gene dissemination and the complex mobility of *optrA* among *Enterococcus* species.

These results underscore the genetic diversity of *optrA*-positive *Enterococcus* isolates in South Korea, spanning multiple host species, sequence types, and genomic contexts, which may facilitate the widespread dissemination of linezolid resistance determinants in both human and animal populations.

### 2.3. Genetic Environment and Phylogenetic Relatedness

**Figure 3:**
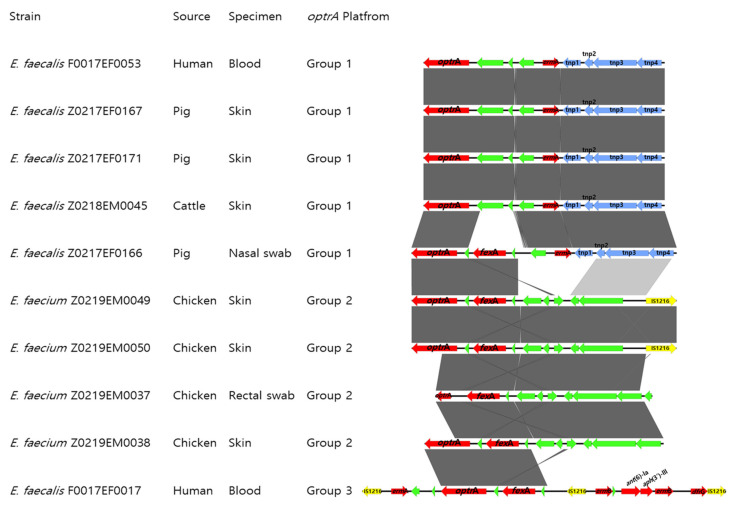
BLAST-based alignment of the genetic environments surrounding the *optrA* gene in ten *Enterococcus* isolates. Resistance genes are shown as red arrows, and insertion sequences (ISs) as yellow arrows. Transposase genes and hypothetical proteins are depicted in blue and green, respectively. Arrowheads indicate the direction of transcription. Shaded grey regions represent nucleotide identity >99% across aligned segments. The visualization was generated using Easyfig v2.2.5 to illustrate homologous regions and structural differences among the isolates based on draft genome assemblies.

The genetic environments surrounding the *optrA* gene were classified into three distinct groups based on structural features and associated mobile genetic elements (Figure 3).

Group 1 included five *E. faecalis* isolates obtained from hospitalized patients (ST476), pigs (ST256), and cattle (ST256). In these isolates, *optrA* or *optrA-fexA* segments were located on the chromosome adjacent to the transposon Tn6674, which harbored conserved transposase genes (*tnpA*, *tnpB*, and *tnpC*). Tn6674 is known to be an active transposable element that can excise from the chromosome and form circular intermediates capable of integrating into new genomic locations [11,12].

Group 2 comprised four *E. faecium* isolates from chickens (ST12, ST124, and ST446). These carried a chromosomal *optrA*-*fexA*-*xerC* segment, which showed 99.96% nucleotide identity and 40% coverage to a pig-derived plasmid, pP47-61 (GenBank accession no. CP91102) [13], suggesting a possible plasmid-to-chromosome transfer and subsequent adaptation in avian *E. faecium*.

Group 3 consisted of a single clinical *E. faecalis* isolate carrying the *optrA*-*fexA* segment on plasmid pF17EF17. This plasmid exhibited 99.7% similarity to the previously reported multidrug-resistant plasmid pEF123 (GenBank accession no. KX599977) and carried additional resistance genes, including *erm*A, *erm*B, *aph(3’)-IIIa*, *ant(6)-Ia*, *str*, *dfrG*, *tet*M, and *cat* (Supplementary Figure S2). This structure exemplifies the plasmid-mediated accumulation and dissemination of multiple resistance determinants.

To assess phylogenetic relationships, a single nucleotide polymorphism (SNP)-based analysis was conducted by comparing the genomes of the ten *optrA*-positive isolates from this study with publicly available *Enterococcus* genomes harboring *optrA* from various global sources. This analysis revealed considerable genetic diversity among both *E. faecalis* and *E. faecium* isolates. Notably, one clade of ST476 *E. faecalis* isolates clustered closely with strains isolated from humans in South Korea, chickens in China, and retail chicken products in Europe, indicating potential global dissemination of this lineage via the food chain or zoonotic transmission routes (Supplementary Figures S3 and S4).

## 3. Discussion

In this study, we investigated the prevalence and genetic characteristics of LRE isolated from humans and livestock in South Korea, focusing on the genetic environments of the *optrA* gene. Our findings indicate that, while the overall linezolid resistance rate in clinical isolates remained low (0.2%), livestock isolates exhibited a significantly higher prevalence (2.4%), particularly among pigs (3.3%) and cattle (4.3%). This disparity underscores the differential selection pressures between human and animal populations, potentially influenced by increased florfenicol use in Korean livestock production. This pattern aligns with previous national reports, according to which florfenicol use has nearly doubled over the past decade [14], potentially driving cross-resistance selection between phenicols and oxazolidinones.

Compared to previous studies, our data indicate significantly lower prevalence rates. Hu et al. (2022), who analyzed clinical isolates from a single tertiary care hospital in South Korea, reported higher rates of linezolid resistance (2.4%) and *optrA* carriage (1.3%) [15]. The lower rates observed in our study likely reflect the broader scope and more representative nature of nationwide surveillance compared to single-center studies.

In Vietnam, Ha et al. (2023) reported substantially higher rates of both linezolid resistance and *optrA* positivity in livestock, particularly in chickens (37.3%) and pigs (11.3%) [16]. Such elevated prevalence in animal reservoirs suggests the potential role of antimicrobial use in agriculture in promoting resistance dissemination, although specific usage patterns in these regions require further investigation. Similarly, Roy et al. (2025) described linezolid resistance and *optrA* positivity rates of 2.2% in clinical isolates from Bangladesh [17], considerably higher than our clinical data.

Reports from Europe and the United States provide additional context. In Belgium, *optrA*-positive *E. faecalis* strains were identified as key drivers of linezolid resistance across multiple sequence types, reflecting the gene’s dissemination within human clinical settings [18,19]. In the United States, hospital outbreaks involving linezolid- and vancomycin-resistant *E. faecium* carrying *optrA* have been increasingly documented, particularly since 2020 [20]. A recent nationwide sentinel surveillance study by Kent et al. (2024) further demonstrated the presence of *optrA* in clinical *E. faecium* isolates from diverse geographic regions, including those carried on linear plasmids co-harboring *vanA*, underscoring the potential for dissemination via mobile genetic elements [21].

Collectively, our findings suggest that, while *optrA*-mediated linezolid resistance remains relatively uncommon in South Korea, especially in human clinical isolates, higher prevalence rates in animal sources warrant continued surveillance. Notably, the *optrA*-positive linezolid resistance rate further demonstrates the divergence between host populations. These data emphasize that, despite low overall resistance rates, the presence of *optrA* in both human and livestock populations is non-negligible and warrants vigilant surveillance.

The detection of plasmid-mediated *optrA* in a clinical *E. faecalis* isolate represents a significant public health concern. Although plasmid-borne *optrA* was observed in only one human isolate, it was situated within a multidrug resistance plasmid harboring genes conferring resistance to aminoglycosides, macrolides, and tetracyclines. This finding highlights the potential for horizontal gene transfer and co-selection in clinical settings.

An intriguing observation was the host-specific distribution of *optrA* gene variants. Variants *optrA_5* and *optrA_20* were exclusive to human isolates, *optrA_7* predominated in pigs and cattle, and *optrA_15* was restricted to chicken isolates. Although the phenotypic consequences of these allelic variations require further investigation, their consistent host-specific distribution suggests possible host-adaptive evolution or independent acquisition events shaped by distinct ecological reservoirs and antimicrobial selection pressures. These patterns may also reflect the influence of region-specific farming practices on the genetic diversification of *optrA* variants. Variations in antibiotic usage protocols, livestock density, biosecurity practices, and feed regimens are likely to create distinct ecological niches that drive microevolutionary changes. In particular, strong antimicrobial selection pressure in intensive farming environments may promote the emergence and persistence of specific *optrA* alleles. Moreover, limited animal movement between farms or species could further reinforce variant segregation, leading to the development of host-associated lineages with unique resistance profiles.

Phylogenetic analysis further provided compelling evidence of zoonotic potential and international dissemination. While most isolates displayed considerable phylogenetic diversity, *E. faecalis* ST476 isolates from humans in South Korea were genetically related to isolates from chickens and retail chicken products in other countries. This finding is congruent with recent global studies identifying ST476 as a widely disseminated lineage harboring *optrA*, reported across diverse hosts—including humans, livestock, and wild birds—in Europe, Asia, and North America [22,23,24,25]. Brenciani et al. (2024) identified ST476 *optrA*-positive isolates from humans and animals across 15 countries [22]. Similar findings were reported in Italy from wild peregrine falcons [23], and in Scotland across clinical and agricultural settings [24]. While direct U.S. ST476 reports are limited, *optrA*-positive *E. faecalis* strains have been identified in U.S. surveillance [25].

These findings collectively highlight the zoonotic potential and international spread of *optrA*-carrying ST476 lineages, reinforcing the critical need for integrated One Health antimicrobial resistance surveillance frameworks. The co-occurrence of *optrA* and *fexA* within both chromosomal Tn6674 elements and plasmids may facilitate dissemination across bacterial species and host boundaries. Furthermore, increasing florfenicol use in livestock could exert indirect selection pressure for linezolid resistance through the co-selection of phenicol–oxazolidinone resistance determinants.

Therefore, continuous, integrated surveillance, applying a One Health approach, is urgently required to monitor the emergence and dissemination of linezolid resistance and associated genes across both human and animal populations. Future studies should investigate the functional impacts of *optrA* gene variants, their transferability, and the ecological drivers shaping their distribution.

## 4. Materials and Methods

### 4.1. Bacterial Isolates

A total of 2156 *Enterococcus* isolates were retrospectively analyzed, comprising 1409 clinical isolates (536 *E. faecalis* and 873 *E. faecium*) collected through the Korean Global Antimicrobial Resistance Surveillance System (Kor-GLASS) [26], and 747 isolates (606 *E. faecalis* and 141 *E. faecium*) from healthy animals collected through a nationwide survey of the prevalence of antimicrobial-resistant bacteria in livestock between 2017 and 2019. Briefly, livestock farms in eight provinces across the country were randomly selected for sample collection. Each animal sample was collected from at least 10 different points (nasal, skin, rectal, and fecal swabs).

### 4.2. Screening of Linezolid Resistance Genes

All *Enterococcus* isolates with a linezolid minimum inhibitory concentration (MIC) >4 mg/L were screened for the presence of *cfr* and *optrA* using real-time PCR. The following primers and probe sets were used: for *optrA*-F, (5′-ATTTTATCGAGGAATTAAGCTTTTGAC-3′), *optrA*-R (5′-AGCGTCAGTTAAAGTAGATAATTTAAG-3′), and *optrA*-P (FAM 5′-CTGCAACAATCACGCCCAATTTCG-3′); and for *cfr*, *cfr*-F (5′-TTCTTTTATGGGAATGGGTGAAG-3′), *cfr*-R (5′-CGTAAACGAATCAAGAGCATCAA-3′), and *cfr*-P (FAM-5′-TCTAGCCAACCGTCAAG-3′).

### 4.3. Whole-Genome Sequencing and Analysis

All *optrA*-positive isolates were subjected to whole-genome sequencing using the Illumina NovaSeq and PcaBio Sequel platforms. Genomic DNA was isolated using the Wizard Genomic DNA Purification Kit (Promega, Madison, WI, USA) according to the manufacturer’s instructions. For short-read sequencing, libraries were prepared using the TruSeq Nano DNA Library Prep kit (Illumina, San Diego, CA, USA) and sequenced as paired-end reads (2 × 150bp) using the Illumina Novaseq platform. Long-read sequencing libraries were prepared using SMRTbell Express Template Prep Kit 2.0 (Pacific Biosciences, MenloPark, CA, USA) and subsequently sequenced using PacBio Sequel I (Pacific Biosciences). Hybrid assemblies were generated using Unicycler v.0.4.8 [27] using default settings. Genes were annotated using Prokka v.1.14.6 [28], and multi-locus typing (MLST) was determined using the in silico mlst tool (v.2.21.0, https://github.com/tseemann/mlst, accessed on 1 December 2024). Antimicrobial resistance genes were identified using ResFinder 4.3.1 (https://genepi.food.dtu.dk/resfinder, accessed on 1 December 2024) and the Comprehensive Antibiotic Resistance Database (CARD) 3.1.1 [29]. Reads were also screened for mutations in 23S rRNA genes associated with linezolid resistance using LRE-finder 1.0. Allele numbers for *optrA* were assigned according to the scheme described by Freitas et al. [11]. Phylogenetic relatedness was determined using kSNP3 v3.1 [30]. The optimal k-mer size was determined using the Kchooser program, available with the package. A phylogenetic tree was visualized using Micoreact [31] with the respective metadata.

## 5. Conclusions

In this nationwide surveillance study, the prevalences of linezolid resistance and *optrA*-positive *Enterococcus* isolates in both clinical and livestock sources in South Korea were found to be relatively low compared to in other countries, particularly Southeast Asia. However, higher rates in livestock populations and the detection of multidrug-resistant plasmid-borne *optrA* in clinical isolates underscore the potential for horizontal gene transfer and co-selection. The identification of host-specific *optrA* variants and the phylogenetic clustering of *E. faecalis* ST476 isolates from humans and animals with international strains highlight the zoonotic potential and global dissemination risk of these lineages. These findings address the key aims of this study and provide valuable insights into the molecular epidemiology and dissemination of *optrA*-mediated resistance.

Our findings emphasize the importance of continuous molecular surveillance using a One Health approach to monitor the emergence and spread of transferable linezolid resistance genes. Further research should focus on understanding the ecological factors driving *optrA* distribution, the functional impact of different *optrA* variants, and the dynamics of resistance gene transfer across human, animal, and environmental reservoirs.

## Figures and Tables

**Table 1:** Phenotypic and genotypic characteristics of *optrA*-positive isolates.

Strain	Isolation (Data, Location)	Origin	Specimen	ST	*van* Operon	Acquired LinezolidResistance Gene			23S rRNA Mutation	MIC (mg/L)					
						*optrA* ^a^	*cfr*	*poxtA*		LZD	VAN	TEC	CHL	DAP	TGC
*E. faecalis* E0017EF0053	December 2017, Jeolla	Human	Blood	476	-	+ (c)	-	-	-	8	1	<0.125	32	1	0.025
*E. faecalis* F0017EF0017	July 2017, Gyeongsang	Human	Blood	330	-	+ (p)	-	-	-	8	1	<0.125	64	2	0.25
*E. faecalis* Z0217EF0166	September 2017, Jeolla	Pig	Nasal swab	256	-	+ (c)	-	+	-	>16	1	<0.25	16	1	0.25
*E. faecalis* Z0217EF0167	September 2017, Jeolla	Pig	Skin	256	-	+ (c)	-	+	-	>16	2	<0.25	>64	1	0.25
*E. faecalis* Z0217EF0171	September 2017, Jeolla	Pig	Skin	256	-	+ (c)	-	+	-	>16	1	<0.25	>64	2	0.25
*E. faecalis* Z0218EM0045	Jun 2018, Gyeongsang	Cattle	Skin	256	-	+ (c)	-	-	-	8	1	<0.25	>64	1	0.25
*E. faecium* Z0219EM0037	April 2019, Jeolla	Chicken	Rectal swab	12	-	+ (c)	-	-	-	8	1	<0.25	64	4	0.125
*E. faecium* Z0219EM0038	April 2019, Jeolla	Chicken	Skin	124	-	+ (c)	-	-	-	8	0.5	<0.25	32	4	0.125
*E. faecium* Z0219EM0049	May 2019, Gyeongsang	Chicken	Skin	446	-	+ (c)	-	-	-	8	1	<0.25	64	4	0.125
*E. faecium* Z0219EM0050	May 2019, Gyeongsang	Chicken	Skin	446	-	+ (c)	-	-	-	8	1	0.5	64	4	0.125

LZD, linezolid; VAN, vancomycin; TEC, teicoplanin; CHL, chloramphenicol; DAP, daptomycin; TGC, tigecycline. ^a^ (c), chromosome-encoded; (p), plasmid-mediated.

## Data Availability

All data generated or analyzed during this study are included in this published article and its Supplementary Materials. Raw sequencing reads generated in this study were registered in the NCBI for Biotechnology Information BioProject database under Project No. PRJNA935058.

## Supplementary Materials

The following supporting information can be downloaded at: https://www.mdpi.com/article/10.3390/antibiotics14060571/s1, Figure S1. Representation of the alignment of relevant SNPs in the different *optrA* variants. The variant names used are the same as those included in the CGE database until *optrA_19* and new variants were attributed from *optrA_20*. Figure S2. Schematic diagram of the structural organization of plasmid pF17EF17 from *E. faecalis* F0017EF0017, isolated from a human in this study and previously reported as a pEF123 plasmid (GenBank acc. no. KX599977). Genes of interest and their orientations are represented by arrows as follows: red indicates antibiotic resistance genes, yellow indicates Iss, and blue indicates rep genes. Figure S3. Phylogenetic tree containing optrA-carrying E. faecalis. Blue indicates strains used in this study. Figure S4. Phylogenetic tree containing *E. faecium*. Black square indicates *optrA*-carrying strains. Shaded region indicates strains used in this study.
