# Supplementary material for: Molecular Epidemiology and Genetic Context of optrA-Carrying Linezolid-Resistant Enterococci from Humans and Animals in South Korea
Source: Antibiotics (Basel). 2025 Jun 3;14(6):571. doi: 10.3390/antibiotics14060571 (PMC12189147; doi:10.3390/antibiotics14060571)
Supplement: Supplementary file 1 [file antibiotics-14-00571-s001.zip › antibiotics-3662314-supplementary.pdf]

Supplementary data

Variant	Strain	GenBank	Reference (PMID)	Position																								
				7	34	240	311	335	366	392	405	406	475	527	583	607	650	740	767	850	895	1036	1042	1050	1179	1185	1187	1198
optrA_1	E349	KP399637	25977397	A	A	G	T	C	C	-	A	T	C	T	A	C	A	G	G	C	T	G	A	C	G	T	G	T
optrA_2	E016, 05K173	KT862781	26903276	G	A	G	T	C	C	-	A	T	C	G	A	C	A	G	G	T	T	G	A	C	G	T	G	T
optrA_3	XY17	KT862780	26903276	A	A	G	T	C	C	-	A	T	C	T	A	C	A	G	G	C	T	G	A	C	G	T	G	T
optrA_4	E419, 13F074	KT862777	26903276	A	A	G	T	A	C	-	A	T	C	G	A	C	A	G	G	C	T	G	A	C	G	T	G	T
optrA_5	E147	KT862783	26903276	A	A	G	T	C	C	-	A	T	C	T	A	C	A	G	G	C	T	G	A	C	G	T	G	T
optrA_7	G20, 09L128	KT862784	26903276	G	A	G	T	C	C	-	A	T	C	G	A	C	A	G	G	T	T	G	A	C	A	T	G	T
optrA_8	10-2-2, 09P292	KT862775	26903276	A	A	G	T	C	C	-	A	T	C	G	A	C	A	G	G	C	T	G	A	C	G	T	G	T
optrA_11	E079, 11C219	KT862782	26903276	A	A	G	T	C	C	-	A	T	C	G	A	C	A	G	G	C	T	G	A	C	A	T	G	T
optrA_13	E35048	KT892063	26702919	G	T	A	T	C	A	-	G	G	T	G	T	T	A	G	G	C	C	A	G	T	A	C	T	C
optrA_14	wo28-3	KT601170	26953332	G	T	G	T	C	C	-	A	T	C	G	A	C	A	G	G	C	T	G	A	C	A	T	G	T
optrA_15	13G340	KX620936	27645239	G	A	G	T	C	C	-	A	T	C	G	A	C	A	G	G	T	T	G	A	C	G	T	G	T
optrA_16	11C233	KX620941	27645239	A	A	G	T	A	C	-	A	T	C	G	A	C	A	G	G	C	T	G	A	C	G	T	G	T
optrA_17	13G339	KX620934	27645239	A	A	G	T	C	C	-	A	T	C	G	A	C	A	G	A	C	T	G	A	C	G	T	G	T
optrA_18	11L002	KX620942	27645239	A	A	G	G	C	C	-	A	T	C	G	A	C	A	G	A	C	T	G	A	C	G	T	G	T
optrA_19	11R356	KX620939	27645239	G	T	A	T	C	C	-	A	T	C	G	A	C	A	A	G	T	T	G	A	C	A	T	G	T
optrA_5		E17EF53		A	A	G	T	C	C	-	A	T	C	T	A	C	A	G	G	C	T	G	A	C	G	T	G	T
optrA_20		F17EF17		A	A	G	T	A	C	A	A	T	C	G	A	C	A	G	G	C	T	G	A	C	G	T	G	T
optrA_7		Z0217EF166		G	A	G	T	C	C	-	A	T	C	G	A	C	A	G	G	T	T	G	A	C	A	T	G	T
optrA_7		Z0217EF167		G	A	G	T	C	C	-	A	T	C	G	A	C	A	G	G	T	T	G	A	C	A	T	G	T
optrA_7		Z0217EF171		G	A	G	T	C	C	-	A	T	C	G	A	C	A	G	G	T	T	G	A	C	A	T	G	T
optrA_7		Z0218EM45		G	A	G	T	C	C	-	A	T	C	G	A	C	A	G	G	T	T	G	A	C	A	T	G	T
optrA_15		Z0219EM38		G	A	G	T	C	C	-	A	T	C	G	A	C	A	G	G	T	T	G	A	C	G	T	G	T
optrA_15		Z0219EM49		G	A	G	T	C	C	-	A	T	C	G	A	C	A	G	G	T	T	G	A	C	G	T	G	T
optrA_15		Z0219EM50		G	A	G	T	C	C	-	A	T	C	G	A	C	A	G	G	T	T	G	A	C	G	T	G	T
Truncated		Z0219EM37		G	A	G	T	C	C	-	A	T	C	G	A	C	-											

Variant	Strain	GenBank	Reference (PMID)	Position																								
				1442	1450	1465	1492	1501	1526	1531	1534	1588	1622	1627	1655	1663	1679	1687	1694	1738	1780	1834	1841	1867	1880	1900	1920	1949
optrA_1	E349	KP399637	25977397	A	T	A	A	T	C	T	A	A	C	T	A	T	A	A	C	C	G	C	G	A	A	T	A	A
optrA_2	E016, 05K173	KT862781	26903276	A	T	A	A	T	C	T	A	A	C	T	A	T	A	A	C	C	G	C	G	G	A	T	A	A
optrA_3	XY17	KT862780	26903276	A	T	A	A	T	C	T	T	A	C	T	A	T	A	A	C	C	G	C	G	A	A	T	A	A
optrA_4	E419, 13F074	KT862777	26903276	A	T	A	A	T	C	T	A	A	C	T	A	T	A	A	C	C	A	T	G	A	A	T	A	A
optrA_5	E147	KT862783	26903276	A	T	A	A	T	C	T	A	A	C	T	A	T	A	A	C	C	A	T	G	A	A	T	A	A
optrA_7	G20, 09L128	KT862784	26903276	A	T	A	A	T	C	T	A	A	C	T	A	T	A	A	C	C	A	T	G	A	A	T	A	A
optrA_8	10-2-2, 09P292	KT862775	26903276	C	T	A	A	T	C	T	A	A	C	T	A	T	A	A	C	C	G	C	G	A	A	T	A	A
optrA_11	E079, 11C219	KT862782	26903276	A	T	A	A	T	C	T	A	A	C	T	A	T	A	A	C	C	G	C	G	A	A	T	A	A
optrA_13	E35048	KT892063	26702919	A	A	G	C	C	A	C	A	G	G	C	T	C	T	T	A	T	G	C	C	A	T	A	T	G
optrA_14	wo28-3	KT601170	26953332	A	T	A	A	T	C	T	A	A	C	T	A	T	A	A	C	C	G	C	G	A	A	T	A	A
optrA_15	13G340	KX620936	27645239	A	T	A	A	T	C	T	A	A	C	T	A	T	A	A	C	C	G	C	G	A	A	T	A	A
optrA_16	11C233	KX620941	27645239	C	T	A	A	T	C	T	A	A	C	T	A	T	A	A	C	C	G	C	G	A	A	T	A	A
optrA_17	13G339	KX620934	27645239	A	T	A	A	T	C	T	A	A	C	T	A	T	A	A	C	C	G	C	G	A	A	T	A	A
optrA_18	11L002	KX620942	27645239	A	T	A	A	T	C	T	A	A	C	T	A	T	A	A	C	C	G	C	G	A	A	T	A	A
optrA_19	11R356	KX620939	27645239	A	T	A	A	T	C	T	A	A	C	T	A	T	A	A	C	C	G	C	G	G	A	T	A	A
optrA_5		E17EF53		A	T	A	A	T	C	T	A	A	C	T	A	T	A	A	C	C	A	T	G	A	A	T	A	A
optrA_20		F17EF17		A	T	A	A	T	C	T	A	A	C	T	A	T	A	A	C	C	A	T	G	A	A	T	A	A
optrA_7		Z0217EF166		A	T	A	A	T	C	T	A	A	C	T	A	T	A	A	C	C	A	T	G	A	A	T	A	A
optrA_7		Z0217EF167		A	T	A	A	T	C	T	A	A	C	T	A	T	A	A	C	C	A	T	G	A	A	T	A	A
optrA_7		Z0217EF171		A	T	A	A	T	C	T	A	A	C	T	A	T	A	A	C	C	A	T	G	A	A	T	A	A
optrA_7		Z0218EM45		A	T	A	A	T	C	T	A	A	C	T	A	T	A	A	C	C	A	T	G	A	A	T	A	A
optrA_15		Z0219EM38		A	T	A	A	T	C	T	A	A	C	T	A	T	A	A	C	C	G	C	G	A	A	T	A	A
optrA_15		Z0219EM49		A	T	A	A	T	C	T	A	A	C	T	A	T	A	A	C	C	G	C	G	A	A	T	A	A
optrA_15		Z0219EM50		A	T	A	A	T	C	T	A	A	C	T	A	T	A	A	C	C	G	C	G	A	A	T	A	A
Truncated		Z0219EM37																										

Figure S1. Representation of the alignment of relevant SNPs in the different *optrA* variants. The variant names used are the same as those included in the CGE database until *optrA_19* and new variants were attributed from *optrA_20*.

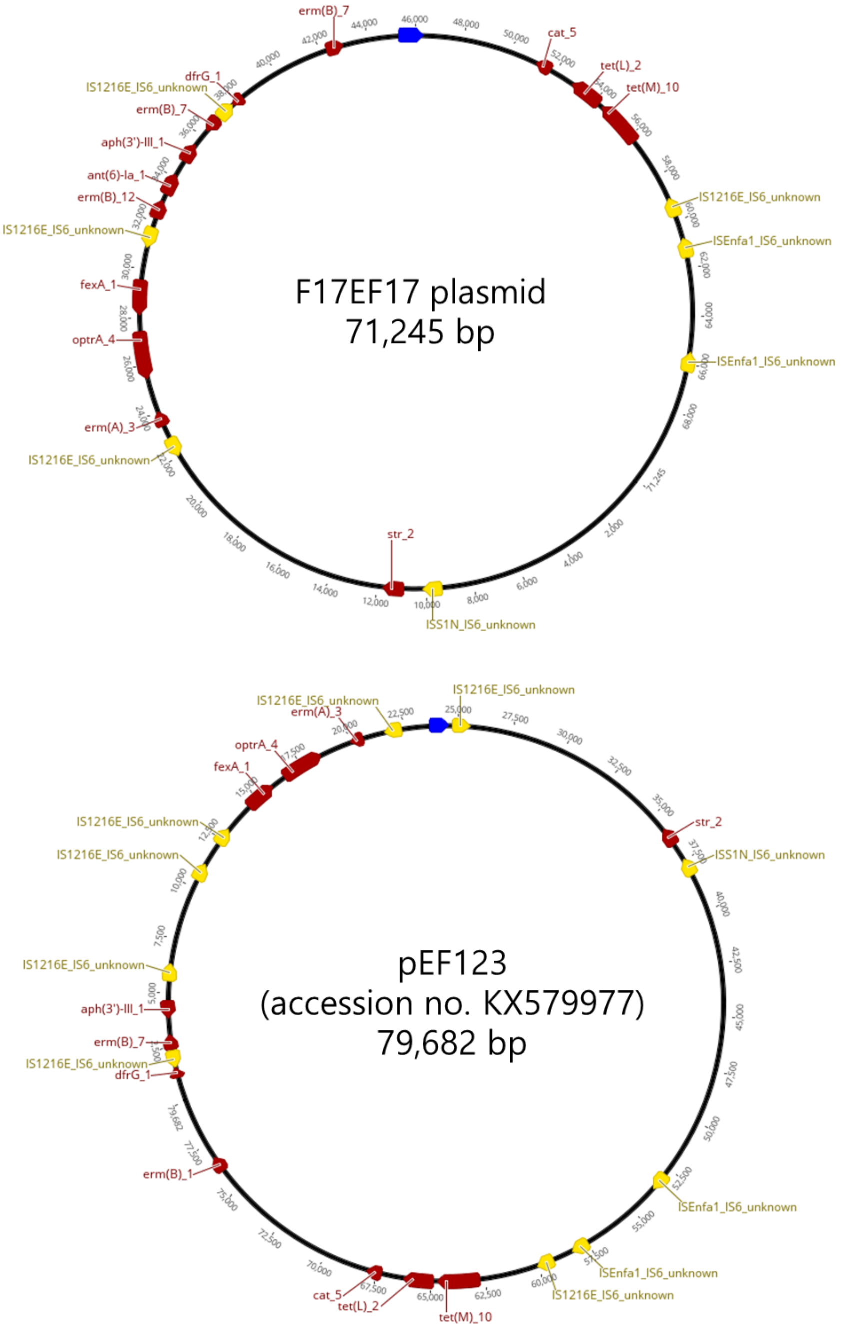

Figure S2. Schematic diagram of the structural organization of plasmid pF17EF17 from *E. faecalis* F0017EF0017 isolated from human in this study and previously reported pEF123 plasmid (GenBank acc. no. KX599977). Genes of interest and their orientation are represented by arrows as follows: red indicates antibiotic resistance gens, yellow indicates Iss, and blue indicates rep gene.

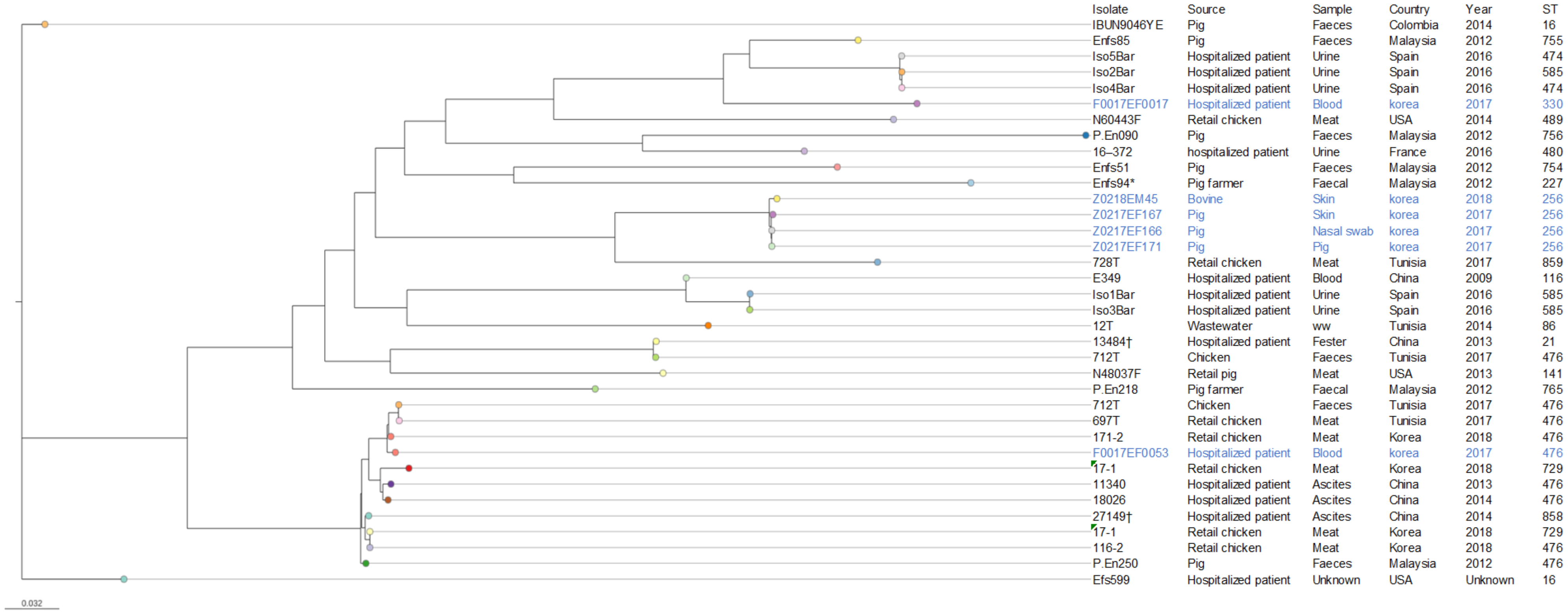

Figure S3. Phylogenetic tree containing *optrA*-carrying *E. faecalis*. Blue indicates strains used in this study.

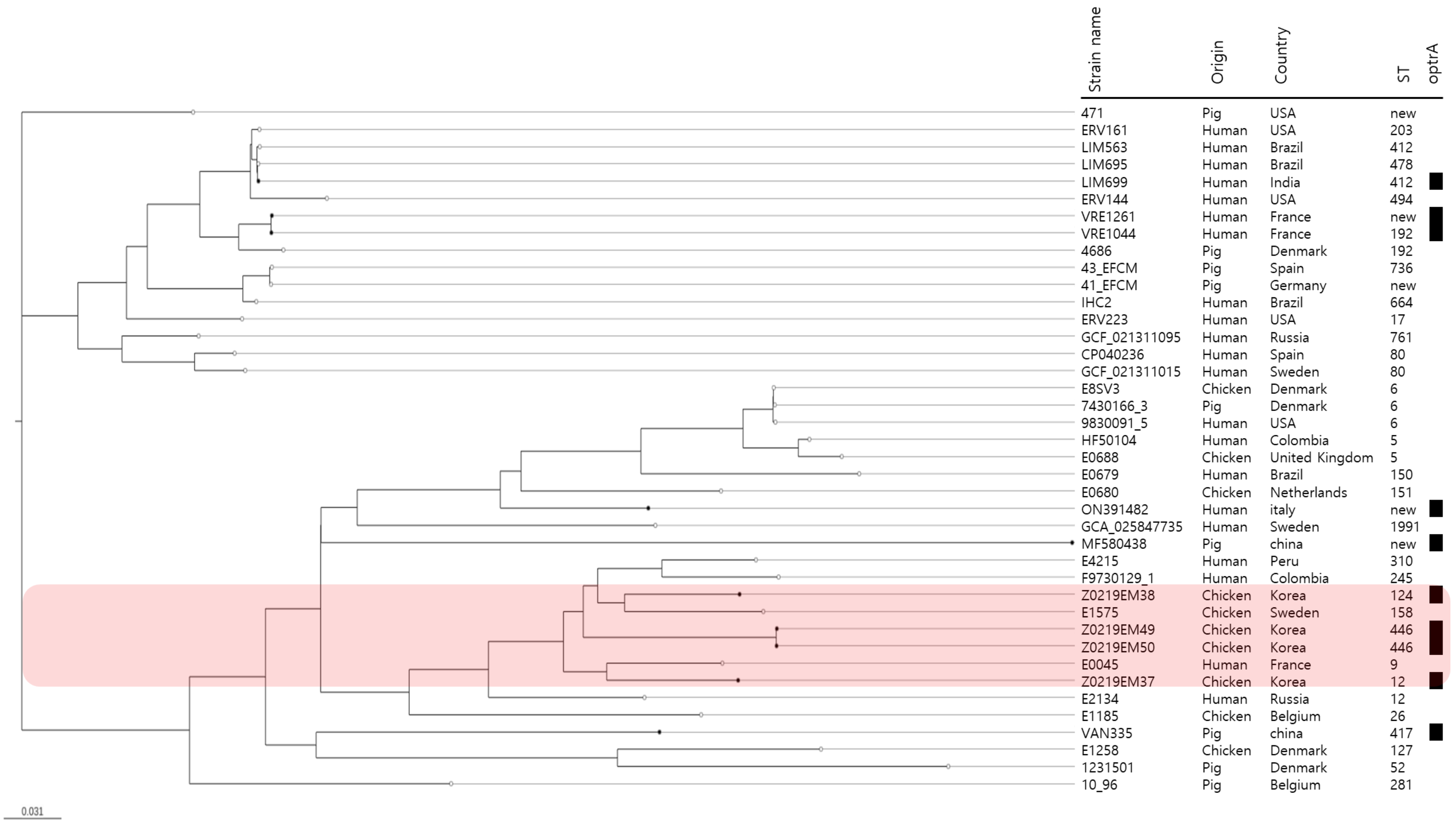

Figure S4. Phylogenetic tree containing *E. faecium*. Black square indicates *optrA*-carrying strains. Shading region indicates strains used in this study.
